# Bronzed Skin

**Published:** 1856-06

**Authors:** 


### BRONZED SKIN AND DISEASE OF THE SUPRA-RENAL CAPSULES.

In our number for September, 1855, we recorded a case of *change of color of the skin from white to that of the mulatto*, without any well-defined cause to which it could be attributed, or any discovered post mortem lesion with which it could be associated. We were not aware until quite recently that there was any supposed connection between this condition of the skin and a special morbid state of any organ; and have been interested in the record of cases in some late numbers of the *London Medical Times and Gazette*, *London Lancet*, and *Association Medical Journal*, intended to illustrate "the connection between bronzed skin and disease of the *supra-renal* capsules," to which attention has been directed by Dr. Addison, the Senior Physician of Guy's Hospital, in a monograph on the "Diseases of the Supra-renal Capsules," recently published. According to the views of Dr. A., which are the result of clinical experience and are supported by eleven carefully narrated cases, among other symptoms indicative of disease of these organs, is "peculiar browning or bronzing of the skin," which may depend upon any disease which affects their disorganization, as cancer, tubercle, abcess. He says, that patients, suffering from this symptom, "fall gradually, and without any obvious cause, into a peculiar form of debility, which results almost invariably in death within a limited period."

The number of cases yet observed, is, of course, too small to establish a decided connection between these symptoms and disease of these organs, but they are sufficient to awaken interest in the subject, and lead to investigation and settlement of the question whether a new form of disease is to be added to the nosological list. The editor of the *Medical Times and Gazette* has commenced a series of cases bearing upon this point, and appeals to the profession for the contribution of facts on the subject.

According to Dr. Addison, there are no marked symptoms of this disease, except progressive loss of strength, and the peculiar change in the color of the skin; but it is a characteristic of the debility attending it, that is rarely attended by much emaciation; and that, though there is much flabbiness of tissue, the subject of it "retains throughout a general bulkiness of frame which contrasts strongly with his extreme feebleness," usually, no other important visceral complication supervenes.

So little importance has been heretofore attached to the supra-renal capsules, that the examination of them has been almost universally neglected, until recently. Since Dr. Addison commenced his researches, attention has been paid to this point; and during the past two years, in five hundred inspections, which have been made at Guy's Hospital, under the observation of Drs. Wilks and Habershon, "only two cases have been met with where disease of these organs was found without having been previously diagnosed;" and in one of these, in which the patient died of cancer, the report mentions that the "patient's face presented a dingy hue."

Anatomists differ as to the appearance and structure of these bodies in the healthy state, and we are entirely ignorant of their physiological functions; and until these points have been settled by a long series of careful observations, no conclusion can of course be drawn as to what changes should be considered as morbid. Dr. Wilks, in a communication on the subject, in the December number of the *Medical Times and Gazette*, has given a wood-cut showing a section of the organ in a state of health, a magnified horizontal section of the cortical structure, etc., etc. Mr. Hutchinson remarks, in speaking of the relation between disease of the supra-renal capsules and the cachexia with bronzed skin, "the very liberal supply of nerves received by the supra-renal organs, leads to the conjecture that they are functionally very closely associated with the sympathetic system." He says that "Bergmann and Kolliker agree in describing their medullary center as little more than a nervous plexus; and the latter has counted no fewer than thirty-three nerve-trunks entering a right supra-renal body." Hence, the strong suspicion that these organs may have some share in lesions of nutrition.

The same journal, in its numbers for December, 1855, and January, 1856, gives us six fatal cases of decided "bronzed skin," in three of which positive disease of the supra-renal capsules was found after death. In a fourth case, reported by Dr. Peacock, in which this peculiar hue of the skin was found associated with great debility, there was not the slightest evidence of disease either in the kidneys or the supra-renal capsules; but in this case a calcareous concretion of the size of a pea was found in the substance of the medulla oblongta, with well-marked softening at that part. In the other two cases, no autopsy was made. In the case reported by us, before referred to, no examination was made of the supra-renal capsules; but the kidneys were reported as normal in structure, though of only about half the usual size. The lesions varied in the three cases which were examined. The ages of the six patients reported in the *Medical Times and Gazette*, were respectively, 12, 14, 24, 24, 27, or 28, and 28 years. The age of our patient was 23 years. Death was sudden, and without any marked change in the symptoms preceding it, in five of these cases; and in the other, the manner of death was not known, as the patient was lost sight of; death was quite sudden in our patient. All of the patients, whose cases are reported in that journal, had walked out or walked about the day previous to death, including the one in which no alteration of the supra-renal capsules was found after death. Three were males, and three females.

In the *Association Medical Journal*, for January 19, 1856, two cases are related by Dr. W. Budd, both females, one 42

and the other 40 years of age, with the same peculiar hue of skin, and the same constitutional symptoms, both of which proved fatal, but in neither of which was any post mortem examination made.

In the number of the same journal for January 26, 1856, Mr. C. M. Thompson gives the post mortem of a case of what he calls "bronzed skin," in a female about 30 years of age, in which "the skin on the whole body was dark bronze; *the palms of the hands were white;* the conjunctivæ were perfectly clear and white." The discoloration had followed upon a fever, and had gradually increased. She had suffered from "severe dyspepsia, loss of appetite, frequent vomiting, emaciation, and also from violent spasm, resembling gall-stone;" but there was no proof of bilious obstruction. "The viscera were all found in a healthy state after death; but the gall-bladder was greatly distended, and *its duct entirely obliterated*, while the hepatic duct was pervious." This writer speaks of having, in the course of his practice, met with four cases of "black jaundice," in all of which he says "the *bronze discoloration* was very great," but there was a great difference between them and the present case; the orange eye and the obstructed bile clearly indicating the nature of the disease in them."

At the meeting of the Pathological Society in London, January 1, 1856, according to the *London Lancet*, January 12th, Mr. Hutchinson exhibited two diseased supra-renal capsules taken from a patient who had died of extreme debility, in which the patient is said to have presented, in a marked degree, the peculiar brown skin regarded by Dr. Addison as diagnostic of disease of these organs; but no particulars of special lesion, or of the history of the case are given, and we are inclined to believe that they are two of the cases more fully reported in the *London Times and Gazette* already referred to.—*N. Y. Medical Times.*

